# Association between mammographic density and basal-like and luminal A breast cancer subtypes

**DOI:** 10.1186/bcr3470

**Published:** 2013-09-04

**Authors:** Hilda Razzaghi, Melissa A Troester, Gretchen L Gierach, Andrew F Olshan, Bonnie C Yankaskas, Robert C Millikan

**Affiliations:** 1 Department of Epidemiology, Gillings Global School of Public Health, University of North Carolina, Chapel Hill, NC, USA; 2 Linberger Comprehensive Cancer Center, University of North Carolina, Chapel Hill, NC, USA; 3 Hormonal and Reproductive Epidemiology Branch, Division of Cancer Epidemiology and Genetics, National Cancer Institute, National Institutes of Health, Rockville, MD, USA; 4 Department of Radiology, School of Medicine, University of North Carolina, Chapel Hill, NC, USA; 5 National Center on Birth Defects and Developmental Disabilities, Centers for Disease Control and Prevention, 1600 Clifton Road, Atlanta, GA 30345, USA

**Keywords:** Mammographic density, Breast cancer, Subtypes, Basal-like, Epidemiology

## Abstract

**Introduction:**

Mammographic density is a strong risk factor for breast cancer overall, but few studies have examined the association between mammographic density and specific subtypes of breast cancer, especially aggressive basal-like breast cancers. Because basal-like breast cancers are less frequently screen-detected, it is important to understand how mammographic density relates to risk of basal-like breast cancer.

**Methods:**

We estimated associations between mammographic density and breast cancer risk according to breast cancer subtype. Cases and controls were participants in the Carolina Breast Cancer Study (CBCS) who also had mammograms recorded in the Carolina Mammography Registry (CMR). A total of 491 cases had mammograms within five years prior to and one year after diagnosis and 528 controls had screening or diagnostic mammograms close to the dates of selection into CBCS. Mammographic density was reported to the CMR using Breast Imaging Reporting and Data System categories. The expression of estrogen receptor (ER), progesterone receptor (PR), human epidermal growth factor receptor 1 and 2 (HER1 and HER2), and cytokeratin 5/6 (CK5/6) were assessed by immunohistochemistry and dichotomized as positive or negative, with ER+ and/or PR+, and HER2- tumors classified as luminal A and ER-, PR-, HER2-, HER1+ and/or CK5/6+ tumors classified as basal-like breast cancer. Triple negative tumors were defined as negative for ER, PR and HER2. Of the 491 cases 175 were missing information on subtypes; the remaining cases included 181 luminal A, 17 luminal B, 48 basal-like, 29 ER-/PR-/HER2+, and 41 unclassified subtypes. Odds ratios comparing each subtype to all controls and case-case odds ratios comparing mammographic density distributions in basal-like to luminal A breast cancers were estimated using logistic regression.

**Results:**

Mammographic density was associated with increased risk of both luminal A and basal-like breast cancers, although estimates were imprecise. The magnitude of the odds ratio associated with mammographic density was not substantially different between basal-like and luminal A cancers in case–control analyses and case-case analyses (case-case OR = 1.08 (95% confidence interval: 0.30, 3.84)).

**Conclusions:**

These results suggest that risk estimates associated with mammographic density are not distinct for separate breast cancer subtypes (basal-like/triple negative vs. luminal A breast cancers). Studies with a larger number of basal-like breast cancers are needed to confirm our findings.

## Introduction

Studies of the molecular profiles of breast cancers have indicated that breast tumors can be classified into five etiologically and prognostically relevant subtypes on the basis of gene expression patterns [1]. Since then luminal A (estrogen receptor (ER)-positive, progesterone receptor (PR)-positive, and human epidermal growth factor receptor (HER)-2/*neu*-negative) and basal-like (ER-negative, PR-negative, HER-2/neu-negative, and cytokeratin 5/6-positive and/or HER-1 positive) breast cancers have been widely studied clinically and epidemiologically [2-14], with luminal A cancers being of interest because they represent the largest percentage (45%) of cancers, and basal-like cancers, whereas rarer (5 to 15% of cases), having the poorest survival outcomes [4,15,16]. Basal-like breast cancers are more prevalent among younger African American women with breast cancer and show unique risk factor patterns, often having risk factor-specific associations in the opposite direction of those for breast cancer overall and luminal A tumors [4,7-14]. For example, the protective effects of parity are observed with breast cancers overall and with luminal breast cancers, but appear to be reversed with basal-like breast cancer [4]. It is important to understand how distinct molecular subtypes are related to established or suspected breast cancer risk factors.

Among breast cancer risk factors, mammographic density is one of the strongest and most consistent risk factors, with studies estimating that women with the highest mammographic density may be at a 4- to 6-fold increased risk of developing breast cancer compared to women with the lowest mammographic density [17-24]. However, there are conflicting results on the association between mammographic density and risk of breast cancer subtypes defined by hormone receptor status (reviewed in Boyd *et al*. [25]). Of the eight case–control and cohort studies examining the association between mammographic density and breast cancer risk by tumor hormonal status, six [26-31] observed increased risk of both ER- positive (ER^+^) and ER-negative (ER^-^) tumors among those with the most dense breast tissue, and two [32,33] observed increased risks for ER+ tumors only. Of the thirteen studies with cases only, all but two [34,35] concluded that there were no significant differences in mammographic density by hormone receptor status [36-46]. A recent meta-analysis on the topic also concluded that mammographic density is similarly strongly associated with both ER^+^ and ER^-^ tumors [47].

Despite these largely negative results, some uncertainty remains. Notably, recent results suggest that basal-like breast cancers are associated with decreased involution of terminal duct lobular units (TDLUs), the structures from which most breast cancer precursors and cancers develop [48]. Because elevated mammographic density is also associated with decreased TDLU involution [49], it may be expected that basal-like breast cancers would therefore be associated with higher mammographic density. However, data relating mammographic density to specific intrinsic subtypes are limited [35]. More detailed subtyping that distinguishes HER2+ tumors from basal-like tumors and from tumors with poor immunohistochemical (IHC) reaction due to fixing artifacts is needed. A few studies have evaluated the association between mammographic density and three IHC markers (ER, PR, HER-2/*neu*), but further resolution of these triple-negative tumors into those that are truly basal-like would improve these analyses [26,30,31,40].

We hypothesized that the association between mammographic density and breast cancer risk would be different for basal-like versus luminal A breast cancers. We therefore examined the association between mammographic density and basal-like and luminal A subtypes of breast cancer using a panel of five IHC markers. Participants in the Carolina Breast Cancer Study (CBCS) were matched to participants in the Carolina Mammography Registry (CMR) to allow estimation of the association between mammographic density and risk of these specific breast cancer subtypes.

## Methods

### Study setting and population

Subjects in this study were participants in the CBCS who also had mammograms recorded in the CMR. CBCS is a population-based, case–control study conducted in 24 counties in North Carolina, designed to identify genetic and environmental factors for breast cancer risk in African Americans and Caucasians. Briefly, CBCS participants were women aged 20 to 74 years; cases were identified from the North Carolina Central Cancer Registry and controls were identified using drivers’ license and Medicare beneficiary lists. Controls were age and race frequency-matched to cases. The CMR, funded by the Department of Defense in 1994 and supported as part of the Breast Cancer Surveillance Consortium by the National Cancer institute since 1995, is a mammography registry that prospectively collects data from women and radiologists in mammography facilities in community practice. Both CBCS and CMR are described in detail in Razzaghi *et al.*[50].

Data from the CBCS and the CMR were combined to allow for case–control and case-case analyses of mammographic density by breast cancer subtype. Briefly, CMR and CBCS were linked using probabilistic linkage with four variables; first and last name, date of birth, and last four digits of the social security number [51-53]. Breast Imaging Reporting and Data System (BI-RADS) breast density, age, and current use of hormone therapy at the time of the mammogram were collected from the CMR, and all other participant data were taken from the CBCS. The following counties from the CBCS were not represented in this study because there were no matching cases and controls in the CMR: Alamance, Orange, Wake, Johnston, Lee, Harnett, Bertie, Wilson, Edgecombe, Pitt, Pamlico, Beaufort, and Tyrell.

### Tumor blocks and immunohistochemistry assays

The details of breast cancer subtyping in CBCS have been published previously [4]. Briefly, all breast cancers underwent pathology review and descriptive data including type of biopsy, tumor size, laterality, and other characteristics were abstracted from pathology reports. Three H&E-stained slides were produced from each of the paraffin blocks when slices were made for molecular and IHC analyses. These slides were reviewed in a standardized fashion by the study pathologist to confirm the diagnosis of breast cancer and to assign histologic classification [54]. The following markers were used to determine breast cancer subtypes: luminal A (ER^+^ and/or PR^+^, HER2^-^), luminal B (ER^+^ and/or PR^+^, HER2^+^), basal-like (ER^-^, PR^-^, HER2^-^, HER1^+^ and/or cytokeratin (CK)5/6^+^), HER2^+^/ER- (ER^-^, PR-, HER2^+^), and unclassified (negative for all five markers) [4,16]. Only luminal A and basal-like cancers are examined in detail in the current analysis due to the small number of HER2+ and luminal B cases.

To determine subtype, tumor blocks were sectioned and stained for a panel of IHC markers at the IHC Core Laboratory, University of North Carolina (UNC). Commercially available antibodies to ER, HER2, HER1, and Cytokeratin 5/6 were used in this study [16,55,56]. For invasive cases, ER/PR status was obtained from medical records for 80% of cases and determined using IHC assays performed at UNC for the remaining cases. For 11% of the cases with missing status for ER/PR on medical records, paraffin-embedded tissues were used and ER/PR status was determined at the UNC laboratory using IHC. ER/PR status was missing for the remaining 9% of the cases [16,54,57].

Of the 491 cases that were in both the CMR and CBCS, 175 had missing information on subtype; the remaining cases included 181 luminal A, 17 luminal B, 48 basal-like, 29 ER^-^/PR^-^/HER2^+^, and 41 unclassified subtypes.

### Mammographic density assessment

Mammographic density was determined by the radiologist at the time of the mammogram and recorded qualitatively in the CMR using the BI-RADS scoring system of the American College of Radiology. BI-RADS density assessment defines four categories of breast tissue composition including: 1) almost entirely fat, 2) scattered fibroglandular densities, 3) heterogeneously dense, and 4) extremely dense [58]. As discussed in Razzaghi *et al.*[50], for cases density was reported from the screening or diagnostic mammogram performed within five years prior to or one year after breast cancer diagnosis. Mammograms for controls were screening or diagnostic mammograms showing no cancer within five years prior to and three years after the selection date. The rationale for choosing a control group with a broader exposure window has been discussed previously [50]. Briefly, studies have shown that elevated risks of breast cancer associated with mammographic density persist for at least 5 years after a mammogram [19,23,59-61]. To assess whether inclusion of diagnostic mammograms for cases where screening mammograms were unavailable affected results, we previously conducted sensitivity analyses. No substantial change in effect estimates for the association between mammographic density and breast cancer risk were observed when cases with only diagnostic mammograms were excluded from analyses [50].

For women with multiple mammograms, the order of preference was (1) the mammogram prior to breast cancer diagnosis or selection date into CBCS with the date closest to diagnosis or selection date and (2) the nearest mammogram after diagnosis/selection. Studies have shown that elevated risks of breast cancer associated with mammographic density persist for at least 5 years, with studies showing lasting effects for 10 years or more for both pre- and postmenopausal women [34,59-61]. Mammograms more than one year following treatment were excluded based on suggestions in the literature that agents used to treat breast cancer may alter mammographic density as early as 18 months after initiating therapy [62]. Mammographic density measured in the CMR is per woman and not per breast. It is expected that mammographic density measured in this way reflects risk because mammographic density is a general marker of breast cancer risk and is not specific to breast side or location of the eventual cancer [63] and because density has been shown to be highly correlated between breasts within a woman [64].

### Statistical analysis

Potential confounders were selected based on prior knowledge and using directed acyclic graphs (DAGs) [65]. We adjusted for age, race, body mass index (BMI), hormone therapy (HT) use, menopausal status, first-degree family history of breast cancer, age at menarche, and parity and age at first full-term pregnancy (with the latter two combined into a single variable). We also adjusted for an offset term used in the CBCS to oversample young African American women [66].

The variable coding schemes were chosen for consistency with previous CBCS publications [4]. As there is substantial biological and epidemiologic heterogeneity between BI-RADS 1 and BI-RADS 2 categories, we did not combine density categories. Rather, we present two models: one uses BI-RADS 1 as the referent group to show the magnitude of effect comparing each category to this lowest risk group, and the other uses BI-RADS 2 as the referent group to increase the stability and/or precision of effect estimates. This sample-coding strategy also facilitates comparisons with our previously published investigation of mammographic density and breast cancer risk [50]. Race was categorized as African American or white based on self-report. Mammographic density was based on the four BI-RADS density categories. Age at diagnosis was used for cases and age at selection into the CBCS for controls and was analyzed as a continuous variable. BMI was calculated as body weight (kg)/height^2^ (m) and was treated as a continuous variable in the analysis. Age at first full-term pregnancy and parity/nulliparity were combined to create a categorical variable that encapsulated both parity status and age at first birth. HT was categorized as current or not-current as collected by the CMR at the time of the mammogram. Because of the association between age, HT use, and mammographic density, we also examined age and current HT use at the time of the mammogram recorded in the CMR, as explained in detail in our previous study [50]. All categorical variables were coded using indicator variables.

We used unconditional logistic regression to estimate the odds ratio (OR) and 95% CI for the association between mammographic density and breast cancer risk (SAS version 9.3, SAS Institute, Cary NC, USA). We considered basal-like and luminal A breast cancers primarily, but we also examined risk of triple-negative breast tumors (ER, PR, and HER-2-negative tumors) to facilitate comparison with previous studies on the association between mammographic density and risk of triple-negative breast cancers. Case-case analyses were used to compare the distribution of mammographic density among patients with basal-like tumors to that among patients with luminal A tumors, and to compare mammographic density among triple-negative patients to luminal A patients. Effect measure modification was not assessed, given the small sample size.

As addressed in our previous study, to assess the comparability of the CMR-CBCS merged data and the full CBCS dataset, we compared the characteristics of participants who matched to the CMR (the current dataset) to those in the entire CBCS by estimating ORs for established breast cancer risk factors. The ORs were similar in the CMR-CBCS merged dataset and the CBCS as a whole for all variables assessed [50].

### Ethical considerations

Both the CMR and the CBCS were approved by the Institutional Review Board of the UNC and were conducted in compliance with the Helsinki Declaration. Specific patient-informed consent was not required for this study, since all women consented to participate in the CBCS and the program was authorized to collect and use health and clinical information from study participants for evaluation and scientific research.

## Results

Characteristics of all breast cancer cases (n = 491) and women with basal-like and luminal A tumors as well as 528 controls are presented in Table 1. Compared with women with luminal A breast cancer, women with the basal-like subtype were younger, had higher BMI and waist-to-height ratio (WHR), were more likely to be African American, premenopausal, younger than 13 years at menarche, parous with first full-term pregnancy at younger than 26 years, not current HT users, users of oral contraceptives, and never having breastfed (Table 1). Thus, associations with standard risk factors showed similar patterns by subtype as reported for the CBCS overall [4].

**Table 1:** Population characteristics by tumor subtype, basal-like and luminal A breast cancers

**Variable**	**Overall cases versus controls**		**Cases**			
	**Cases**	**Controls**	**Basal-like**	**OR (95% CI)**	**Luminal A**	**OR (95% CI)**
Subjects, number	491	528	48		181	
Age (CBCS), mean (range), y^a^	53.2 (28 to 74)	54.0 (31 to 74)	50.2 (33 to 73)	0.99 (0.96, 1.02)	54.5 (31, 74)	1.04 (1.02, 1.06)
BMI, mean (95% CI)	28.6 (15.1, 60.6)	28.8 (14.6, 60.9)	30.9 (19.1 to 44.2)	1.06 (1.02, 1.10)	28.5 (15.0, 52.6)	1.00 (0.98, 1.03)
Number of days, mean (95% CI)^b^	−21 (−1401, 365)	149 (−1617, 1095)	−27 (−938, 365)		−10 (−1050, 365)	
Race, n (%)						
White	297 (60.5%)	324 (61.4%)	21 (43.8%)	1.00	116 (64.1%)	1.00
African American	194 (39.5%)	204 (38.6%)	27 (56.3%)	3.32 (1.80, 6.12)	65 (35.9%)	1.31 (0.90, 1.89)
Menopausal status, n (%)						
Premenopausal	200 (40.7%)	213 (40.3%)	25 (52.1%)	1.00	67 (37.0%)	1.00
Postmenopausal	291 (59.3%)	315 (59.7%)	23 (47.9%)	0.90 (0.49, 1.65)	114 (63.0%)	1.83 (1.27, 2.65)
Family history, n (%)^c^						
No	386 (81.1%)	440 (85.6%)	39 (83.0%)	1.00	149 (84.2%)	1.00
Yes	90 (18.9%)	74 (14.4%)	8 (17.0%)	1.24 (0.55-2.82)	28 (15.8%)	1.11 (0.67-1.84)
Missing	15	14	1		4	
Age at menarche, n (%)						
<13 y	257 (52.3%)	230 (43.6%)	32 (66.7%)	1.00	92 (50.8%)	1.00
≥13 y	234 (47.7%)	298 (56.4%)	16 (33.3%)	0.37 (0.19-0.70)	89 (49.2%)	0.76 (0.53-1.09)
Parity and age at FFTP						
Nulliparous	74 (15.1%)	67 (12.7%)	6 (12.5%)	1.00	31 (17.1%)	1.00
Parous, <26 y	312 (63.5%)	347 (65.7%)	36 (75.0%)	2.07 (1.04-4.15)	107 (59.1%)	0.93 (0.64-1.34)
Parous, 26+y	105 (21.4%)	114 (21.6%)	6 (12.5%)	0.43 (0.18-1.06)	43 (23.8%)	0.96 (0.63-1.47)
Breastfeeding, n (%)						
Never	299 (60.9%)	324 (61.4%)	32 (66.7%)	1.00	110 (60.8%)	1.00
Ever	192 (39.1%)	204 (38.6%)	16 (33.3%)	0.84 (0.44-1.60)	71 (39.2%)	1.09 (0.75-1.57)
Lifetime duration lactation, n (%)						
Never	299 (60.9%)	324 (61.4%)	32 (66.7%)	1.00	110 (60.8%)	1.00
>0-3 months	72 (14.7%)	69 (13.1%)	9 (18.8%)	1.71 (0.77-3.79)	26 (14.4%)	1.14 (0.68-1.92)
4+ months	120 (24.4%)	135 (25.6%)	7 (14.6%)	0.50 (0.22-1.16)	45 (24.9%)	1.02 (0.67-1.55)
Current HT use, n (%)^d^						
Yes	129 (26.4%)	181 (35.0%)	9 (18.8%)	1.00	43 (23.9%)	1.00
No	359 (73.6%)	336 (65.0%)	39 (81.2%)	2.36 (1.11-5.05)	137 (76.1%)	1.84 (1.23-2.77)
Missing	3	11	0		1	
Oral contraceptive use, n (%)						
Never	170 (34.6%)	170 (32.4%)	11 (22.9%)	1.00	72 (39.8%)	1.00
Ever	321 (65.4%)	355 (67.6%)	37 (77.1%)	1.21 (0.59-2.46)	109 (60.2%)	0.49 (0.34-0.71)
Missing	0	3	0		0	
WHR, n (%)						
<0.77	132 (27.3%)	169 (32.3%)	4 (8.7%)	1.00	45 (25.4%)	1.00
0.77 to 0.83	171 (35.3%)	173 (33.0%)	17 (37.0%)	1.19 (0.63-2.24)	69 (39.0%)	1.41 (0.97-2.05)
≥0.84	181 (37.4%)	182 (34.7%)	25 (54.3%)	2.40 (1.30-4.42)	63 (35.6%)	1.17 (0.80-1.71)
Missing	7	4	2		4	

^a^Mean age at diagnosis for cases and selection for controls in the CBCS; ^b^mean number of days between diagnosis date for cases and selection date for controls in the CBCS, and the date of the mammogram chosen to assess mammographic density; ^c^first-degree family history of breast cancer; ^d^current hormone therapy use at the time of the mammogram. OR, odds ratio; CBCS, Carolina Breast Cancer Study; BMI, body mass index; FFTP, first full-term pregnancy; HT, hormone therapy; WHR, waist-to-hip ratio; n, number of subjects.

Table 2 presents the ORs and 95% CIs for adjusted models with both BI-RADS 1 (model 1) and 2 (model 2) as the reference groups. Model 1 is included to facilitate comparison with previous studies that have reported risk for the BI-RADS 4 group who had ‘extremely dense’ breast tissue, relative to the BI-RADS 1 group who had ‘entirely fatty’ breast tissue, but model 2 allows for more precise estimates due to a larger referent group. Among all women, those with extremely dense breasts had an increased risk of breast cancer compared to women with entirely fatty breasts and those with scattered fibroglandular densities (OR 2.45, 95% CI 0.99, 6.09, and OR 1.19, 95% CI 0.72, 1.95, respectively) (Table 2). Model 1 resulted in a stronger positive case–control association between mammographic density and breast cancer risk for the basal-like subtype compared to the luminal A subtype (OR 3.6, 95% CI 0.34, 37.97, and OR 1.98, 95% CI 0.54, 7.34, respectively). These associations were of weaker magnitude when using model 2, and associations were of similar magnitude for the basal-like and luminal A subtypes (OR 1.04, 95% CI 0.34, 3.17, and OR 0.98, 95% CI 0.50, 1.92, respectively) (Table 2). These results suggest no heterogeneity of breast cancer risk according to intrinsic subtype; however, the estimates were generally imprecise as evidenced by the wide confidence intervals.

**Table 2:** Odds ratios (ORs) and 95% confidence intervals (CIs) for breast cancer risk by tumor subtype associated with BI-RADS-measured mammographic density

	**Number of subjects**		**Cases versus controls**			**Triple-negatives versus controls**	
	**Controls**	**Cases**	**Model 1**^**a**^	**Model 2**^**b**^	**TN cases, n**	**Model 1**	**Model 2**
			**OR (95% CI)**	**OR (95% CI)**		**OR (95% CI)**	**OR (95% CI)**
Almost entirely fat	25	13	1.00 (Referent)	0.48 (0.22, 1.08)	1	1.00 (Referent)	0.17 (0.02, 1.43)
Scattered fibroglandular densities	197	183	2.07 (0.93, 4.59)	1.00 (Referent)	31	5.96 (0.70, 50.64)	1.00 (Referent)
Heterogeneously dense	253	232	2.06 (0.92, 4.60)	1.00 (0.7, 1.35)	40	5.83 (0.68, 50.04)	0.98 (0.5, -1.75)
Extremely dense	53	63	2.45 (0.99, 6.09)	1.19 (0.72, 1.95)	12	7.13 (0.74, 68.90)	1.20 (0.49, 2.90)
			*P*_trend_ = 0.24^c^			*P*_trend_ = 0.31	
			**Basal-like versus controls**			**Luminal A versus controls**	
	**BL cases, n**		**Model 1**	**Model 2**	**LA cases, n**	**Model 1**	**Model 2**
			**OR (95% CI)**	**OR (95% CI)**		**OR (95% CI)**	**OR (95% CI)**
Almost entirely fat	1		1.00 (Referent)	0.29 (0.03, 2.51)	4	1.00 (Referent)	0.49 (0.15, 1.59)
Scattered fibroglandular densities	19		3.45 (0.40, 29.90)	1.00 (Referent)	69	2.03 (0.63, 6.59)	1.00 (Referent)
Heterogeneously dense	22		3.03 (0.34, 26.67)	0.88 (0.43, 1.80)	86	2.09 (0.64, 6.79)	1.03 (0.68, 1.56)
Extremely dense	6		3.58 (0.34, 37.97)	1.04 (0.34, 3.17)	22	1.98 (0.54, 7.34)	0.98 (0.50, 1.92)
			*P*_trend_ = 0.67			*P*_trend_ = 0.60	

^a^Model 1 is adjusted for age, race, body mass index, menopausal status, family history of breast cancer, age at menarche, use of hormone therapy, and parity and age at first full-term pregnancy combined, where BI-RADS category 1 (almost entirely fat) is the referent group; ^b^Model 2 is adjusted for the same variables as Model 1 but BI-RADS category 2 (scattered fibroglandular densities) is the referent group; ^c^*P*-value for trend test is based on the likelihood ratio test statistic and is two-sided. The same ordinal model was fit to assess the *P*-value for trend of Model 1 and Model 2. BI-RADS, Breast Imaging Reporting and Data System; TN, triple-negative; BL, basal-like; LA, luminal-A; n, number of subjects.

To facilitate comparisons with previous studies of mammographic density by breast cancer subtype [26,30,39,45], we also examined the association between density and breast cancer risk in case–control analyses using the triple-negative definition of breast cancer. Model 1 resulted in a large, imprecise estimate for risk of triple negative breast cancer, and model 2 resulted in a higher odds ratio than previously observed for basal-like or luminal A breast cancers (OR 1.20, 95% CI 0.49, 2.90) (Table 2). To directly compare basal-like/triple-negative to luminal A breast cancers, we used case-case analyses for model 2 (Table 3). As expected based on case–control analyses, there were no statistically significant differences between basal-like and luminal A, or between triple-negative and luminal A breast cancers (OR 1.08, 95% CI 0.30, 3.84, and OR 1.17, 95% CI 0.41, 3.35, respectively) in relation to mammographic density. However, it is important to note that all of these case-case analyses are imprecise due to small case numbers. Thus, based on these findings, there was no suggestion of etiologic heterogeneity with respect to mammographic density and subtype.

**Table 3:** Odds ratios (ORs) and 95% confidence intervals (CIs) for case-case analyses comparing the association with BI-RADS-measured mammographic density by breast cancer risk subtypes

	**Basal-like versus luminal A**		**Triple-negative versus luminal A**	
	**Model 1**^**a**^	**Model 2**^**b**^	**Model 1**	**Model 2**
	**OR (95% CI)**	**OR (95% CI)**	**OR (95% CI)**	**OR (95% CI)**
Almost entirely fat	1.00 (Referent)	1.05 (0.10, 10.97)	1.00 (Referent)	0.33 (0.03, 3.95)
Scattered fibroglandular densities	0.95 (0.09, 9.90)	1.00 (Referent)	3.05 (0.25, 36.68)	1.00 (Referent)
Heterogeneously dense	0.63 (0.06, 6.65)	0.67 (0.30, 1.49)	2.62 (0.22, 31.62)	0.86 (0.44, 1.67)
Extremely dense	1.02 (0.08, 13.50)	1.08 (0.30, 3.84)	3.57 (0.26, 49.11)	1.17 (0.41, 3.35)
	*P*_trend_ = 0.66^c^		*P*_trend_ = 0.74	

^a^Model 1 is adjusted for age, race, body mass index, menopausal status, family history of breast cancer, age at menarche, use of hormone therapy, and parity and age at first full-term pregnancy combined, where BI-RADS category 1 (almost entirely fat) is the referent group; ^b^Model 2 is adjusted for the same variables as Model 1 but BI-RADS category 2 (scattered fibroglandular densities) is the referent group; ^c^*P*-value for trend test is based on the likelihood ratio test statistic and is two-sided. The same ordinal model was fit to assess the *P*-value for trend of Model 1 and Model 2. BI-RADS, Breast Imaging Reporting and Data System.

## Discussion

Recent findings of decreased involution of terminal duct lobular units (TDLU) surrounding basal-like breast cancers [48] have renewed interest in evaluating the association between mammographic density and subtype-specific breast cancer risk. TDLU involution has been inversely associated with mammographic density [49], leading to the hypothesis that density may be higher among basal-like breast cancers. Previous studies evaluating the relation between mammographic density and breast cancer subtype have not supported this hypothesis, but these studies have had significant potential for outcome misclassification, given the lack of positive markers for basal-like breast cancer [67]. ER-negative tumors are clinically heterogeneous, including HER2-positive, basal-like, and unclassified tumors. Therefore, further stratification of these tumors and identification of basal-like tumors as distinct from triple-negative tumors (where all markers failed to show positivity) could help improve estimates of the true associations. However, even using five markers in case–control analyses, we observed no difference in the association between mammographic density and breast cancer for luminal A, basal-like or triple-negative breast cancers.

Furthermore, our estimates from case-case analysis, which can be interpreted as ratios of ORs between the two subtypes of breast cancer (luminal A and basal-like), directly estimated the relative strength of association between the two breast cancer subtypes and showed no significant difference between basal-like and luminal A or triple-negative and luminal A breast cancers, similar to previous results [25,30,39]. Considering previous case-only studies, eleven of the thirteen studies that examined mammographic density by hormone receptor status concluded that there were no significant differences [36-46]; only four of these studies (all null) examined the association using breast cancer subtypes including the triple-negative subtype [30,40,45,46]. Our previous findings [50] showed that mammographic density was positively associated with breast cancer risk overall; here, the stratified analyses for both luminal A and basal-like breast cancers show similar effect estimates, such that mammographic density is a risk factor for both subtypes with no evidence of heterogeneity by tumor subtype. Using intrinsic subtypes of breast cancer, our findings were largely consistent with the majority of prior studies evaluating the relation between mammographic density and breast cancer risk by molecular subtypes of breast cancer.

It is possible that there are genetic and heritable factors that alter mammographic density and breast cancer risk overall, and are therefore responsible for the association of mammographic density and breast cancer regardless of breast cancer subtype [68]. For example, heritable differences in exposure or response to hormones and growth factors may increase proliferative activity and quantities of stromal and epithelial tissue, with effects on both mammographic density and breast cancer risk across all subtypes [68,69]. Consistent with this, two of fourteen established breast cancer susceptibility loci examined in a recent study contributed to between-woman differences in mammographic density [70]. This finding suggests a model that considers mammographic density as an integrated marker of many different hormonal and non-hormonal influences on breast tissue composition, and is also supported by work examining relationships between mammographic density and non-genetic breast cancer risk factors.

In contrast to mammographic density, many well-established breast cancer risk factors have shown opposite effects on basal-like and luminal A subtypes of breast cancer [4]. For example, Millikan *et al.* identified risk factors for the basal-like subtype, including younger age at diagnosis, higher parity, younger age at first full-term pregnancy, shorter duration of breastfeeding, fewer number of children breastfed, fewer number of months breastfeeding per child, and increased WHR ratio [4]. Many other studies have confirmed similar heterogeneity by anthropometric and reproductive factors [10,71-75]. Because many of these variables that have distinct associations with breast cancer subtypes also impact mammographic density, we might have expected to see differences in the association between mammographic density and breast cancer subtype. For example, young age at first full-term pregnancy is associated with lower mammographic density [76] and a reduction in risk for luminal A breast cancers [17]. However, it appears that mammographic density does not have an association with subtypes that is independent of these factors. In our models that controlled for these as potential confounders, there was no evidence of heterogeneity of the association between mammographic density and breast cancer by subtype.

Major strengths of our study were reduced outcome misclassification through use of five markers to identify breast cancer subtypes (ER, PR, HER2, HER1 and CK5/6) and linkage of established datasets to allow for a relatively large study for assessing this association. However, we note that in the years since the subtyping was performed on CBCS Phase I and Phase II, several improvements have been made to further delineate luminal A and luminal B breast cancers. For example, the classification for luminal B tumors has improved by using the Ki67 index (percentage of Ki67-positive cancer nuclei) [76]. Ideally, these newer markers could be added to improve identification of luminal B in CBCS 1 and 2, but we have emphasized luminal A tumors. Results by Bastien *et al.*[9] show that ER, PR and HER2 staining are relatively homogeneous within luminal A cancers (more than 93% and 94% of luminal A cancers are ER- and PR-positive, respectively, and more than 99% of these tumors are HER2-negative). Therefore, it is unlikely that changes in classification schema would substantially bias the estimates for luminal A reported herein. Moreover, results from Bastien *et al.* also show that standard clinical marker, such as grade, cannot capture the same qualitative information that IHC for Ki-67 would obtain. Therefore, further delineation of luminal B tumors was not conducted in this study. Because of our stratification of breast cancers into many groups, we share a limitation of most studies by molecular subtype, namely, small sample size within strata resulting in imprecise effect-measure estimates for each subtype. In addition, menopausal status or other hormonal exposures may be important in determining the effects of mammographic density on breast cancer risk, but we were underpowered to study effect-measure modification and did not attempt these analyses. Although our study is limited by small sample size, this study is the first to have used molecular subtypes to identify basal-like breast cancers. A pooled analysis or meta-analysis of the association between mammographic density and breast cancer subtypes would provide a larger sample size; however, this will only be possible if future studies differentiate between basal-like and triple-negative breast cancers.

Many recent studies have emphasized etiologic heterogeneity by intrinsic subtype. It is important to recognize that intrinsic subtype classification was greatly influenced by clinical needs and is based on heterogeneity of tumors long after the etiologically relevant window has passed. Many genomic changes occurring late in tumor progression may not be relevant from an etiologic perspective. While some studies have found that there is etiologic heterogeneity, pathogenesis of each subtype is not well-defined and other markers of heterogeneity may be more relevant for a given exposure. For example, tumor characteristics that reflect proliferation or response to DNA damage may be important if the mechanism of density-associated risk is mitogenesis or mutagenesis (as suggested by Martin *et al*. [68]). Alternatively, factors such as hormone receptor status may be more important etiologically than intrinsic subtype.

Future studies of breast cancer subtypes and mammographic density by race are desirable, particularly given that basal-like breast cancers are more prevalent in African American women and appear to have distinct etiology. However, based on current data, there is little evidence to support differences in the effect of mammographic density by breast cancer subtype.

## Conclusions

Using five markers in case–control analyses, we observed no difference in the association between mammographic density and breast cancer for luminal A, basal-like or triple-negative breast cancers. Furthermore, our estimates from case-case analysis, which directly estimated the relative strength of association between the two breast cancer subtypes, showed no significant difference between basal-like and luminal A, or triple-negative and luminal A breast cancers.

## Abbreviations

BI-RADS: Breast Imaging Reporting and Data System; BMI: Body mass index; CBCS: Carolina Breast Cancer Study; CK5/6: cytokeratin 5/6; CMR: Carolina Mammography Registry; DAG: Directed acyclic graph; ER: Estrogen receptor; H&E: Hematoxylin and eosin; HER: Human epidermal growth factor receptor; HT: Hormone therapy; IHC: Immunohistochemical; OR: Odds ratio; PR: Progesterone receptor; TDLU: Terminal duct lobular units; UNC: University of North Carolina; WHR: Waist-to-height ratio.

## Competing interests

The authors declare that they have no competing interests.

## Authors’ contributions

HR designed the study, carried out the analysis, and drafted the manuscript. MAT participated in study design, analysis, and the manuscript draft. GLG participated in study design and provided expertise in mammographic density. RCM participated in study design and provided expertise in breast cancer subtypes. BCY and AFO participated in study design. All authors read and approved the final manuscript.
